# Role of aortic distensibility and stiffness in ascending thoracic aneurysm outcomes

**DOI:** 10.1016/j.xjon.2025.10.013

**Published:** 2025-10-24

**Authors:** Axel Gomez, William Carroway, Nipam Raval, Liang Ge, Marko Boskovski, Elaine E. Tseng

**Affiliations:** Division of Adult Cardiothoracic Surgery, Department of Surgery, University of California San Francisco, and San Francisco VA Health Care System, San Francisco, Calif

**Keywords:** aortic aneurysm, aortic dissection, aortic repair, risk prediction, wall stiffness

## Abstract

**Objective:**

Ascending thoracic aortic aneurysms pose a risk of acute dissection. Although diameter guides elective repair, complications often occur below surgical thresholds. Aortic distensibility and stiffness have been proposed as alternative risk markers. We aimed to evaluate the association of deformation indices with all-cause mortality in patients with dilated aortas and ascending thoracic aortic aneurysms.

**Methods:**

Retrospective study of patients with ascending aortic diameter ≥4.0 cm who had a multiphasic electrocardiogram-gated computed tomography angiogram within 3 years of death or between 2021 and 2024. Diameter- and area-based distensibility and stiffness indices were calculated 3 cm above the annulus using dynamic aortic measurements across the cardiac cycle. Differences by mortality status and aortic valve phenotype were assessed using the Wilcoxon rank-sum test.

**Results:**

We included 319 veterans with median age of 75 years (interquartile range, 7.0 years), of whom 35 (11.0%) died within 3 years. Aortic diameter was larger in the mortality group (4.50 vs 4.40 cm; *P* = .005). Bicuspid aortic valve was present in 12 patients (3.8%). No differences in diameter-based distensibility (0.56 vs 0.64 × 10^−6^ cm^2^/dyne; *P* = .38) or stiffness index (23.5 vs 23.5; *P* = .34) were observed by mortality status. Patients with bicuspid aortic valve had higher unadjusted distensibility (1.23 vs 0.63 × 10^−6^ cm^2^/dyne; *P* = .046) and lower stiffness index (12.5 vs 24.4; *P* = .05) compared with tricuspid valves. Area-based metrics were similar.

**Conclusions:**

Aortic distensibility and stiffness index were not associated with all-cause mortality. Deformation indices varied by valve morphology in unadjusted analyses but were attenuated after adjustment. Further studies are needed to evaluate aortic deformation indices with regard to ascending thoracic aortic aneurysm risk stratification.

## Graphical Abstract

**Figure:**
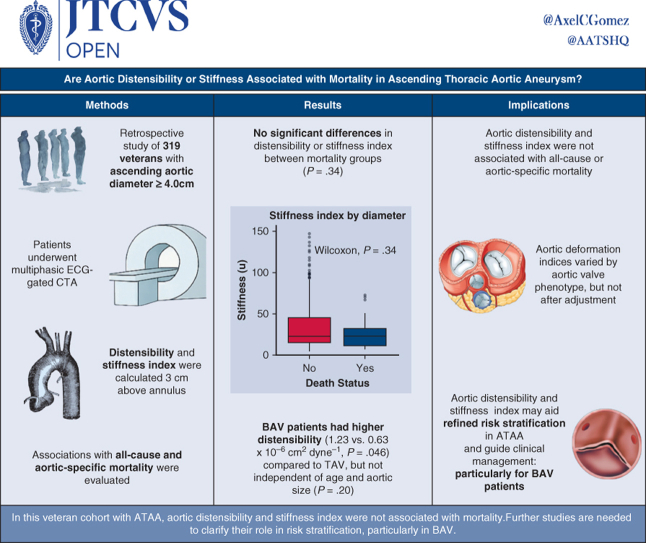

**Figure:**
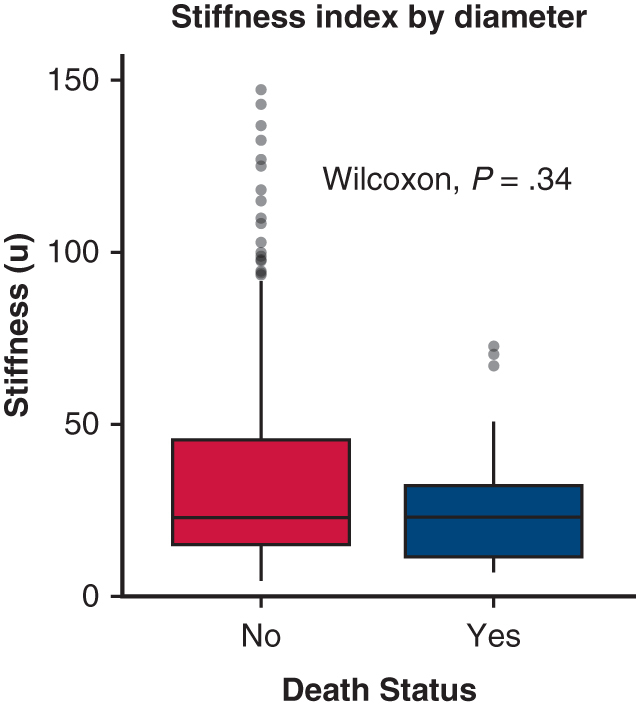
Aortic stiffness was similar in patients with aneurysm with and without all-cause mortality. Dots represent outliers.

**Central Message**

Imaging-derived aortic deformation indices were not associated with all-cause or aortic-specific mortality in veterans with ascending thoracic aortic aneurysms.

**Perspective**

Acute dissection often occurs below current diameter thresholds for aneurysm repair. Aortic deformation indices have been proposed as alternative risk markers. In this veteran cohort, deformation indices did not predict mortality but differed by valve phenotype. Further studies are needed to assess their role in ATAA risk stratification.

Patients with ascending thoracic aortic aneurysm (ATAA) often remain asymptomatic until sudden and catastrophic complications, such as dissection or rupture, occur. Acute aortic dissection is a cardiovascular emergency with a prehospital mortality rate of up to 40%, and perioperative mortality ranging from 10% to 25%.^1^, ^2^, ^3^ Current guidelines recommend prophylactic surgery for aneurysms ≥5.5 cm, or earlier in selected patients with rapid growth, connective tissue disorders, a family history of dissection, or management at specialized centers.^4^ However, aortic diameter alone is an imperfect risk predictor, as up to 60% of dissections occur in aneurysms smaller than 5.5 cm that do not meet surgical criteria.^5^

Improving risk stratification in patients with ATAA remains a critical challenge. Although aortic diameter is used as a surrogate for wall stress via Laplace's Law, it fails to account for the complex geometries and heterogeneous remodeling of the aortic wall.^6^ As a result, there is growing interest in biomechanical markers such as aortic wall strain, distensibility, and aortic stiffness index derived from echocardiography and other imaging modalities.^7^^,^^8^ Prior studies, including work by Emerel and colleagues^7^ and Fortunato and colleagues,^8^ have shown that decreased strain and distensibility, along with increased stiffness, are associated with acute dissection in patients with ATAA. These aortic deformation indices may offer more nuanced and sensitive indicators of wall instability than diameter alone.

In this study, electrocardiogram (ECG)-gated computed tomography angiography (CTA) was used to derive patient-specific measurements of aortic wall strain, distensibility, and stiffness index, accounting for cardiac cycle aortic motion.^9^ We also compared diameter-based and area-based calculation methods to minimize measurement bias inherent in unidimensional assessments. Our objective was to evaluate the relationship between aortic deformation indices and both all-cause and aortic-specific mortality in a large cohort of veterans with dilated aortas and ATAA.

## Methods

### Patient Population

Adult patients with dilated ascending thoracic aortas and ATAAs measuring ≥4.0 cm in diameter, as assessed by CTA, were considered for inclusion. All patients received longitudinal surveillance at the San Francisco Veterans Affairs Healthcare System. We included individuals who underwent ECG-gated CTA with imaging acquired during both systole and diastole. Eligible scans were obtained between January 2021 and November 2024, or within 3 years before patient death or an acute aortic event (eg, dissection, rupture, or intramural hematoma). Patients with a history of aortic events or aortic repair before their ATAA diagnosis were excluded. Patients with connective tissue disease were also excluded. The study was approved by the institutional review boards of the University of California, San Francisco, and San Francisco Veterans Affairs Healthcare System (#10-03594; approval date: September 17, 2010). The requirement for informed consent was waived.

### Clinical Data Collection

Baseline demographics, comorbid conditions, and imaging reports were retrospectively collected from electronic health records. Demographic variables included age, sex, and comorbidities such as hypertension, diabetes, and smoking. Transthoracic echocardiogram (TTE) reports were reviewed to assess aortic valve morphology and valvular disease. Ascending aortic diameter was obtained from CTA reports and represented the maximal diameter of the ascending aorta or aortic root.

Clinical outcomes included all-cause and aortic-specific mortality. Death events were identified using the Veterans Affairs Mortality Data Repository, which integrates Veterans Affairs administrative records with the National Death Index. Aortic-specific mortality was determined through independent review of medical records and California death certificates by 2 senior investigators. This category was defined as deaths directly caused by aortic dissection or rupture, as well as sudden deaths with no alternative attributable cause. Follow-up extended through November 2024.

### Aortic Deformation Measurements

For each patient, aortic wall deformation indices were assessed using their most recent ECG-gated CTA scan. Prior studies demonstrating the association between aortic stiffness index or distensibility with acute type A aortic dissection used measurements 3 cm above the aortic annulus by echocardiography.^8^ Therefore, in this study, maximal diameter and cross-sectional area of the ascending aorta were measured 3 cm above the aortic annulus using centerline analysis for consistency. Measurements were taken during both systolic and diastolic phases using the DICOM viewer Horos version 4.0.1 (Nimble Co). Systolic blood pressure and diastolic blood pressure from the closest clinical encounter to the imaging date were collected. Aortic wall strain, distensibility, and stiffness index were calculated using both diameter-based and area-based measurements.

(1) Aortic wall strain (%)

$$\text{Diameter}\;\text{strain}=100\left(Diameter_{sys}-Diameter_{dias}\right)/Diameter_{dias}$$

$$\text{Area}\;\text{strain}=100\left(A{rea}_{sys}-A{rea}_{dias}\right)/A{rea}_{dias}$$

(2) Distensibility

$$\text{Diameter}\;\text{distensibility}=2\left(Diameter_{sys}-Diameter_{dias}\right)/\;\left(Diameter_{dias}\times \left(SBP-DBP\right)\right)$$

$$\text{Area}\;\text{distensibility}=2\left(A{rea}_{sys}-A{rea}_{dias}\right)/\left(A{rea}_{dias}\times \left(SBP-DBP\right)\right)$$

(3) Stiffness index

$$\text{Diameter}\;\text{stiffness}\;\text{index}=\ln\left(SBP/DBP\right)\;\times \;Diameter_{dias}/\;\left(Diameter_{sys}-Diameter_{dias}\right)$$

$$\text{Area}\;\text{stiffness}\;\text{index}=\ln\left(SBP/DBP\right)\;\times \;A{rea}_{dias}/\left(A{rea}_{sys}-A{rea}_{dias}\right)$$

### Statistical Analysis

Baseline characteristics were summarized as medians with interquartile ranges (IQRs) for continuous variables and as counts with percentages for categorical variables. Comparisons by death status were made using the Wilcoxon rank-sum test for continuous variables and the χ^2^ test of independence for categorical variables. Aortic wall strain, distensibility, and stiffness index—using both diameter-based and area-based measurements—were compared across groups stratified by all-cause mortality. Similar analyses were performed stratified by aortic-specific mortality and by aortic valve phenotype. The Wilcoxon rank-sum test was used for all binary group comparisons. Pearson correlation coefficients were calculated to assess the linear association between baseline aortic diameter and each deformation index. Multivariable linear regression was used to assess the association between aortic valve phenotype and deformation indices, adjusting for baseline aortic diameter and age. All statistical analyses were performed using R version 2024.04.2 (R Foundation for Statistical Computing).

## Results

### Patient Characteristics

Table 1 summarizes baseline patient characteristics for the overall study population and stratified by survival outcome. All patients included had at least 1 ECG-gated CTA scan within 3 years of a mortality event, if applicable. We included 319 patients diagnosed with dilated aortas and ATAA. The median age was 75 years (IQR, 7.0 years), and the median maximal ascending aortic diameter was 4.40 cm (IQR, 0.40 cm); 21 patients (6.6%) had diameters ≥5.0 cm, including 4 patients (1.3%) with diameters ≥5.5 cm. This veteran cohort was predominantly male (98.7%). Common comorbidities included hypertension (81.2%), dyslipidemia (69.0%), and chronic obstructive pulmonary disease (33.2%). A significant smoking history was present in 221 of 319 patients (69.5%). Bicuspid aortic valve (BAV) was identified in 12 patients (3.8%). Baseline characteristics were generally similar between patients with and without a mortality event. However, there were nonsignificant trends toward increased age (76 vs 75 years; *P* = .09) and higher chronic obstructive pulmonary disease prevalence (48.6% vs 31.3%; *P* = .06) in patients who died. Notably, there was a statistically significant difference in ATAA diameter with a marginally larger diameter in the mortality group (4.50 vs 4.40 cm; *P* = .005).

**Table 1:** Baseline population characteristics by survival outcome

Characteristic	Overall	Alive	Death	*P* value
n	319 (100)	284 (89.0)	35 (11.0)	
Age (y)	75.00 [7.00]	75.00 [7.00]	76.00 [10.00]	.09
Diameter (cm)	4.40 [0.40]	4.40 [0.40]	4.50 [0.45]	.005
Male sex	313 (98.7)	280 (98.9)	33 (97.1)	.91
BMI	28.20 [7.40]	28.30 [7.20]	26.35 [12.03]	.29
Race				.63
American Indian/Alaska Native	4 (1.3)	1 (2.9)	3 (1.1)	
Asian	9 (2.8)	0 (0.0)	9 (3.2)	
Native Hawaiian or Other Pacific Islander	7 (2.2)	1 (2.9)	6 (2.1)	
Black or African American	30 (9.4)	2 (5.7)	28 (9.9)	
White	223 (69.9)	28 (80.0)	195 (68.7)	
More than one race	5 (1.6)	0 (0.0)	5 (1.8)	
Unknown/not reported	41 (12.9)	3 (8.6)	38 (13.4)	
Hispanic ethnicity	15 (4.7)	14 (4.9)	1 (2.9)	.90
History smoking	221 (69.5)	194 (68.6)	27 (77.1)	.40
Baseline comorbidities				
CAD	155 (48.6)	141 (49.6)	14 (40.0)	.37
MI	29 (9.1)	27 (9.5)	2 (5.7)	.67
CHF	51 (16.0)	42 (14.8)	9 (25.7)	.16
Hypertension	259 (81.2)	228 (80.3)	31 (88.6)	.34
Dyslipidemia	220 (69.0)	193 (68.0)	27 (77.1)	.36
DM	79 (24.8)	68 (24.0)	11 (31.4)	.45
PVD	21 (6.6)	17 (6.0)	4 (11.4)	.39
CVA	43 (13.5)	36 (12.7)	7 (20.0)	.35
CKD	29 (9.1)	23 (8.1)	6 (17.1)	.15
COPD	106 (33.2)	89 (31.3)	17 (48.6)	.06
OSA	89 (28.0)	80 (28.3)	9 (25.7)	.91
Liver disease	66 (20.7)	59 (20.8)	7 (20.0)	1.00
Aortic stenosis	37 (11.6)	31 (10.9)	6 (17.1)	.42
Aortic insufficiency	64 (20.1)	57 (20.1)	7 (20.0)	1.00
BAV	12 (3.8)	12 (4.2)	0 (0.0)	.44
AAA	10 (3.1)	9 (3.2)	1 (2.9)	1.00

Values are presented as n (%) or median [IQR]. *BMI*, Body mass index; *CAD*, coronary artery disease; *MI*, myocardial infarction; *CHF*, congestive heart failure; *DM*, diabetes mellitus; *PVD*, peripheral vascular disease; *CVA*, cerebrovascular accident; *CKD*, chronic kidney disease; *COPD*, chronic obstructive pulmonary disease; *OSA*, obstructive sleep apnea; *BAV*, bicuspid aortic valve; *AAA*, abdominal aortic aneurysm.

### Association of Aortic Deformation Indices and Mortality

**Figure 1:**
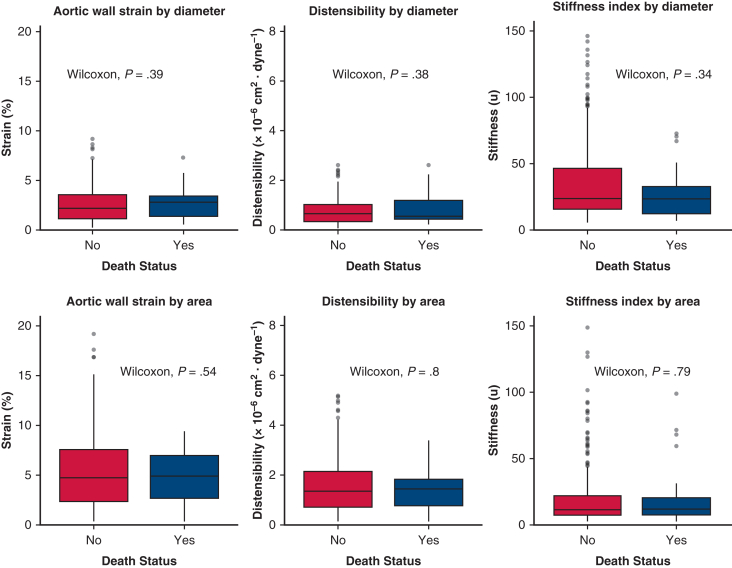
Aortic deformation indices by all-cause mortality status. Boxplots display diameter-based (*top row*) and area-based (*bottom row*) aortic wall strain, distensibility, and stiffness index in patients with and without all-cause mortality. Each plot shows the median (*horizontal line*), interquartile range (*box*), whiskers representing the minimum and maximum nonoutlier values, and outliers (*dots*).

All-cause mortality occurred in 35 of 319 patients (11.0%). There was no significant association between all-cause mortality and diameter-based aortic deformation indices, including strain (2.21 [2.40%] vs 2.79 [2.05%]; *P* = .39), distensibility (0.64 [0.69] × 10^−6^ cm^2^/dyne vs 0.56 [0.78] × 10^−6^ cm^2^/dyne; *P* = .38), or stiffness index (23.5 [30.6] vs 23.5 [20.5]; *P* = .34). Similarly, area-based measures—strain (4.71 [5.22] vs 4.86 [4.26%]; *P* = .54), distensibility (1.32 [1.43] vs 1.41 [1.07] × 10^−6^ cm^2^/dyne; *P* = .80), and stiffness index (11.1 [14.6] vs 11.5 [12.5]; *P* = .79)—also showed no significant differences by all-cause mortality status (Figure 1).

**Figure E1:**
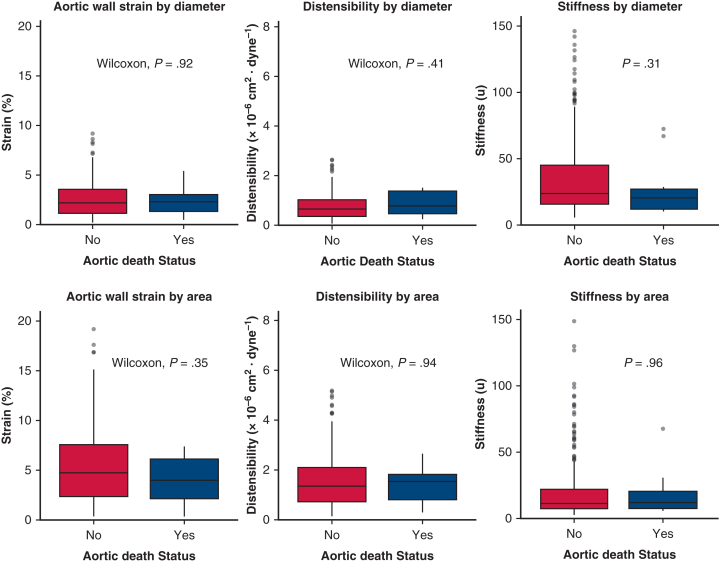
Aortic deformation indices by aortic-specific mortality status. Boxplots display diameter-based (*top row*) and area-based (*bottom row*) aortic wall strain, distensibility, and stiffness index in patients with and without aortic-specific mortality. Each plot shows the median (*horizontal line*), interquartile range (*box*), whiskers representing the minimum and maximum non-outlier values, and outliers (*dots*).

Aortic-specific mortality occurred in 11 of 319 patients (3.4%). All events were sudden unexplained deaths, with no imaging-confirmed dissections or ruptures. There was no significant association between aortic-specific mortality and diameter-based deformation indices, including strain (2.23 [2.37%] vs 2.34 [1.71%]; *P* = .92), distensibility (0.63 [0.69] × 10^−6^ cm^2^/dyne vs 0.76 [0.94] × 10^−6^ cm^2^/dyne; *P* = .41), or stiffness index (23.8 [30.0] vs 20.6 [15.4]; *P* = .31). Similarly, area-based measures—strain (4.74 [5.17%] vs 3.99 [4.02%]; *P* = .35), distensibility (1.32 [1.40] × 10^−6^ cm^2^/dyne vs 1.52 [1.03] × 10^−6^ cm^2^/dyne; *P* = .94), and stiffness index (11.2 [14.6] vs 11.2 [12.2]; *P* = .96)—also showed no significant differences by aortic-specific mortality (Figure E1).

### Association of Aortic Deformation Indices and Aortic Valve Phenotype

**Figure 2:**
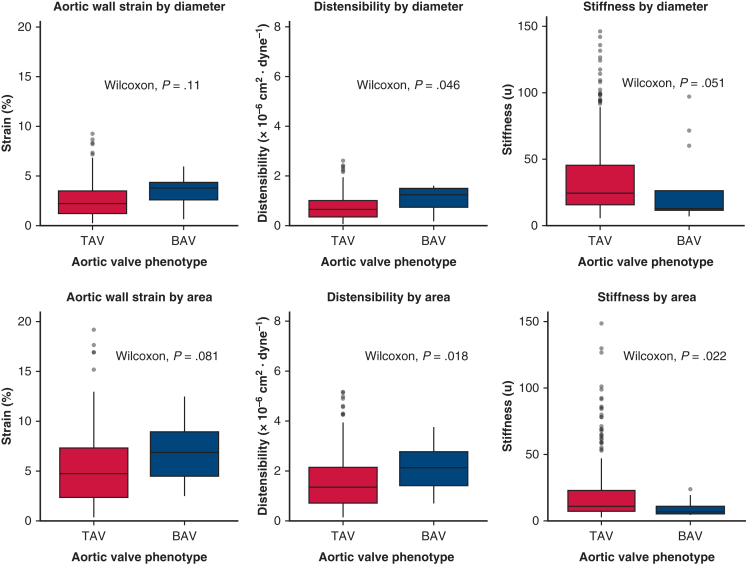
Aortic deformation indices by aortic valve phenotype. Boxplots display diameter-based (*top row*) and area-based (*bottom row*) aortic wall strain, distensibility, and stiffness index in patients with tricuspid aortic valve (*TAV*) and bicuspid aortic valve (*BAV*). Each plot shows the median (*horizontal line*), interquartile range (*box*), whiskers representing the minimum and maximum nonoutlier values, and outliers (*dots*).

Diameter- and area-based distensibility and stiffness index differed between patients with BAV and tricuspid aortic valve (TAV). Diameter-based distensibility was higher in BAV compared with TAV (1.23 [0.76] × 10^−6^ cm^2^/dyne vs 0.63 [0.69] × 10^−6^ cm^2^/dyne; *P* = .046), as was area-based distensibility (2.13 [1.39] × 10^−6^ cm^2^/dyne vs 1.31 [1.40] × 10^−6^ cm^2^/dyne; *P* = .02). Stiffness index trended lower in BAV than TAV for diameter-based measures (12.5 [15.1] vs 24.4 [29.5]; *P* = .05), whereas area-based stiffness was significantly lower in BAV (6.9 [6.0] vs 11.3 [16.5]; *P* = .02). In contrast, no significant differences were observed in strain between BAV and TAV for either diameter-based (3.75 [1.82] vs 2.20 [2.28]; *P* = n .11) or area-based (6.55 [4.55] vs 4.66 [5.08]; *P* = .08) measurements. These findings are illustrated in Figure 2. Median ATAA size was larger in BAV than TAV (4.88 ± 0.25 cm vs 4.40 ± 0.40 cm; *P* < .001), and BAV patients were also younger (65 [21] years vs 75 [6] years; *P* < .001). After adjustment for baseline aortic diameter and age, BAV was no longer significantly associated with diameter-based distensibility (β = 0.22; *P* = .20), area-based distensibility (β = 0.57; *P* = .09), diameter-based stiffness index (β = −6.96; *P* = .50), or area-based stiffness index (β = −12.6; *P* = .14).

### Correlation of Aortic Deformation With ATAA Diameter

**Figure 3:**
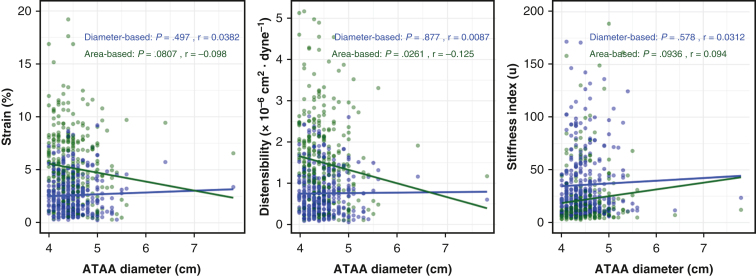
Correlation of aortic deformation indices with ascending thoracic aortic aneurysm (*ATAA*) diameter. Scatterplots show diameter-based (*blue*) and area-based (*green*) aortic wall strain (*left*), distensibility (*center*), and stiffness index (*right*) plotted against ATAA diameter. Pearson correlation coefficients (*r*) and corresponding *P* values are displayed for each relationship. Trend lines represent linear regression fits.

Area-based distensibility demonstrated a weak negative linear correlation with baseline ATAA diameter (*r* = −0.125; *P* = .03), whereas diameter-based distensibility showed no significant correlation (*r* = 0.009; *P* = .88). Wall strain was not significantly correlated with ATAA diameter in either diameter-based (*r* = 0.038; *P* = .50) or area-based (*r* = −0.098; *P* = .08) measurements. Similarly, stiffness index was not significantly correlated with ATAA diameter using either diameter-based (*r* = 0.031; *P* = .58) or area-based (*r* = 0.094; *P* = .09) methods. Scatter plots with linear regression lines are shown (Figure 3).

## Discussion

Despite earlier recognition and improved surveillance, patients with ATAA continue to experience acute dissection or rupture each year.^10^ These events carry high perioperative mortality, ranging from 10% to 25%.^1^, ^2^, ^3^ Current surgical guidelines rely on aortic diameter thresholds to balance the risks of dissection against operative mortality. The American College of Cardiology/American Heart Association recommends elective surgical repair for ATAAs ≥5.5 cm, with earlier intervention considered in the presence of rapid aneurysm growth, connective tissue disorders, family history of dissection, or care at expert aortic centers.^4^ These guidelines are similar between bicuspid and tricuspid aortic valves. The timing of ATAA repair remains controversial due to limited longitudinal data on its natural history. Most available evidence derives from observational studies that are constrained by long latency periods between diagnosis and adverse aortic events, the relatively low incidence of adverse aortic events, and substantial loss to follow-up.^11^, ^12^, ^13^ Moreover, up to 60% to 90% of acute type A dissections occur in aneurysms <5.5 cm that do not meet surgical criteria.^5^ Although maximal diameter is associated with dissection risk, it fails to account for complex aortic geometries and hemodynamic forces and does not reliably predict adverse outcomes across the spectrum of aneurysm sizes. Imaging-derived aortic deformation markers, such as aortic wall strain, distensibility, and stiffness, may offer additional insight into aneurysm vulnerability and hold potential to improve risk stratification beyond diameter alone.

**Figure 4:**
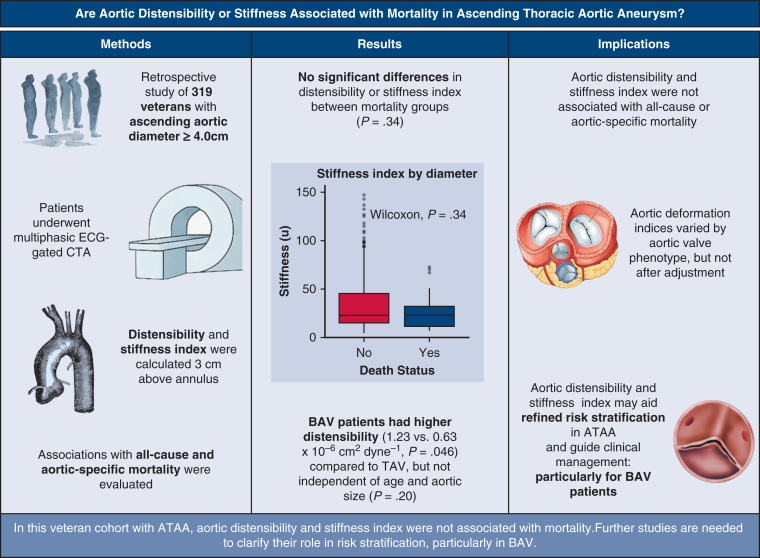
Study schematic demonstrates assessment of aortic distensibility and stiffness index in veterans with ascending thoracic aortic aneurysm (*ATAA*) using electrocardiogram-gated computed tomography imaging. No significant differences in deformation indices were observed by mortality status, but differences were found by aortic valve phenotype. *TAV*, Tricuspid aortic valve; *BAV*, bicuspid aortic valve.

A recent study by Fortunato and colleagues^8^ reported that TTE-derived aortic stiffness was significantly higher in patients with sporadic ATAAs who subsequently experienced an acute dissection, compared with those who did not. That cohort was well matched in terms of aortic diameter and baseline demographics, strengthening the association. These findings suggested that TTE-derived aortic stiffness may serve as a promising biomarker for dissection risk. In related work, the same group also reported reduced TTE-derived strain and distensibility in patients who experienced acute dissection.^7^ Each of those studies relied on measurements 3 cm above the aortic annular plane. In the present study, summarized in Figure 4, we extended prior work by evaluating the relationship between CTA-derived aortic wall deformation and mortality in a cohort of veterans with dilated ascending aortas or ATAAs. We used ECG-gated CTA to ensure that systolic and diastolic measurements were obtained in the same orthogonal plane, 3 cm distal to the aortic annulus, thereby minimizing the influence of cardiac motion during the imaging cycle.^9^ In our analysis, we observed no meaningful differences in aortic stiffness, distensibility, or wall strain between patients who later died from all-cause or aortic-specific mortality and those who did not. Thus, in our cohort of predominantly 4 to 5 cm ATAAs, imaging-derived aortic deformation markers did not discriminate risk of aortic-related mortality. There are important differences between other studies and ours.^7^^,^^8^ Emerel and colleagues^7^ and Fortunato and colleagues^8^ used echocardiography-based measurements and a case-control design that included 22 confirmed dissections and 163 nondissected aneurysmal controls, demonstrating a strong association of dissection with reduced distensibility and increased stiffness. By contrast, our retrospective study was designed to evaluate all-cause and aortic-specific mortality, and no patients with imaging-confirmed acute dissection met inclusion criteria. Accordingly, our study addresses a distinct clinical outcome—mortality—rather than dissection risk. Within our cohort, aortic-specific mortality reflected sudden unexplained deaths, where imaging-confirmed dissections or ruptures were not identified.

The primary methodological difference between echocardiography and CT-based distensibility relates to the precise ability of CT to assess exactly 3 cm above the annular plane in both systole and diastole. In contrast, TTE measurements at this location may be influenced by through-plane motion in the longitudinal direction, thereby capturing a mix of circumferential and longitudinal deformation. Although operator-dependent,^9^ TTE can still yield reproducible plane selection when referenced to the aortic valve. Our inability to detect an association between circumferential distensibility and aortic-specific mortality may reflect that CT-derived measurements do not inherently account for longitudinal through plane motion. Notably, we have previously demonstrated that peak longitudinal stress correlates with all-cause mortality.^14^ It would be worthwhile understanding whether echocardiographic distensibility accounts biomechanically for longitudinal distensibility as well. Our analysis focused solely on circumferential distensibility and wall stiffness, without accounting longitudinal components. These methodological differences may underlie variability in findings between TTE- and CT-based studies. Taken together, our results indicate that circumferential distensibility is not associated with mortality, whereas the contribution of longitudinal distensibility remains uncertain. Future research should evaluate whether longitudinal distensibility, alone or in combination with circumferential indices, can improve risk stratification for dissection or mortality in ATAA.

Prior observational studies have consistently demonstrated that dilated ascending aortas exhibit reduced distensibility and increased stiffness compared to non-dilated aortas. Tjahjadi and colleagues^15^ applied vascular deformation mapping to multiphasic ECG-gated CTAs and found that sporadic aneurysmal aortas had approximately 40% lower distensibility than nondilated aortas. This reduction was observed across the proximal ascending aorta near the sinotubular junction, the midascending aorta, and the proximal arch—with the lowest values noted proximally, the same location used in our study. However, the pattern of distensibility in nondilated aortas has varied across studies.^15^^,^^16^ The relationship between aortic deformation indices and ATAA outcomes remains less well established. Emerel and colleagues’^7^ group was among the first to report associations between decreased distensibility, reduced strain, and increased stiffness in patients with ATAA who later experienced acute dissection. In a small predissection TTE study of n = 7 ATAAs, they observed a 40% reduction in distensibility and a 90% increase in stiffness compared to matched controls.^7^ In another small follow-up study of 22 patients, they reported a 70% to 90% increase in stiffness in predissection ATAAs relative to BAV and TAV controls.^8^ Their univariable logistic regression model demonstrated strong predictive ability for dissection, with an area under the curve of 0.93. By contrast, our larger cohort was designed to evaluate mortality, and we found no significant differences in circumferential distensibility or stiffness index between patients who experienced all-cause or aortic-specific mortality and those who did not.

Nonetheless, the concept of distensibility and stiffness having prognostic potential with regard to aortic-specific mortality or all-cause mortality may still have merit. Dilated aortas and ATAA account for a very small percentage of the normal population. Distensibility and stiffness may have greater predictive ability of mortality within a broader context that includes a normal control population but less discriminatory ability in the subpopulation of dilated aortas and ATAA. The Multi-Ethnic Study of Atherosclerosis population included 5098 individuals without baseline cardiovascular disease, so dilated aortas and ATAA were a miniscule proportion. magnetic resonance imaging/magnetic resonance angiography was used to assess ascending aortic distensibility.^17^ Over 8.5 years of follow-up, lower distensibility was associated with increased all-cause mortality (hazard ratio, 2.7; *P* = .008) and cardiovascular events (hazard ratio, 2.2; *P* = .04). The study by Redheuil and colleagues,^17^ although differing in imaging modality and population, shared a similar end point—all-cause mortality. They also had much longer follow-up to detect differences as well as an inherently different population without cardiovascular disease.

We observed that patients with ATAA with BAV exhibited higher unadjusted distensibility and lower stiffness compared to those with TAV. However, these differences were no longer significant after adjustment for baseline diameter and age, suggesting that the unadjusted findings were largely explained by confounding. The relatively small number of patients with BAV in our cohort further limited statistical power to detect adjusted differences. Prior reports have been inconsistent.^18^, ^19^, ^20^, ^21^, ^22^ For example, Nistri and colleagues^18^ used echocardiography to assess aortic root biomechanics in 128 BAV aneurysms and 114 TAV controls, finding a 36% reduction in distensibility and a 2-fold increase in stiffness in patients with BAV. However, their results were unadjusted, and their BAV group had larger diameters than controls, with stiffness positively correlated with diameter. In contrast, Pascaner and colleagues^23^ conducted a case-control study of 19 patients with BAV and 18 patients with TAV with ATAA and no valvular disease, using 4-dimensional flow magnetic resonance imaging, and found no significant differences between groups. Of note, the study by Fortunato and colleagues^8^ demonstrated nonsignificant trends toward higher distensibility (*P* = .07) and lower stiffness (*P* = .12) in patients with BAV compared with patients with TAV. Our findings are more closely aligned with these latter reports because we did not observe independent differences after adjustment. Heterogeneity across studies may reflect differences in patient populations, imaging modalities, and anatomical sites of measurement. Although our comparison by valve phenotype was a secondary analysis, prior work has linked lower distensibility in BAV to faster aneurysm growth,^21^ and increased likelihood of aortic surgery, independent of other traditional cardiovascular risk factors.^24^ BAV-ATAA guidelines no longer espouse earlier operation than TAV-ATAA and the lower stiffness index and higher distensibility of BAV- than TAV-ATAA support those recommendations. These discrepant findings underscore the need for larger, prospective studies to clarify the role of deformation indices in BAV-associated ATAA.

### Limitations

This study was conducted in a cohort of US veterans, a population that is predominantly male, older, and with a high burden of comorbidities, which may limit the generalizability of our findings to broader populations, in particular women and younger patients. Additionally, we did not observe significant associations between aortic deformation indices and mortality outcomes. Distensibility and stiffness index values were nearly identical across mortality groups, with no apparent trends. Although the study may have been underpowered to detect small differences, it is unlikely that moderate or large differences were missed. Blood pressure is dynamic, and the measurements used may not have reflected the exact state at the time of CT imaging, which could have influenced the calculation of distensibility and stiffness indices. Because our cohort did not include any patients with imaging-confirmed dissection, we were unable to evaluate this outcome directly. Despite these negative findings, our results contribute to the understanding of sporadic ATAA and help reduce publication bias in the field of aortic biomechanics. Our diameter measurements were derived from ECG-gated CTA, which allowed us to minimize motion artifacts and standardize the measurement plane. All measurements were taken 3 cm distal to the aortic annulus, consistent with prior studies.^7^^,^^8^ However, distensibility is known to vary along the aorta,^15^ and measurements taken at alternative locations—such as the site of maximal cross-sectional area—may provide greater discriminatory power.

## Conclusions

In this cohort of patients with sporadic ATAA, aortic distensibility and stiffness index were not associated with all-cause or aortic-specific mortality. Although patients with BAV had higher unadjusted distensibility and lower stiffness compared with patients with TAV, these differences were no longer significant after adjustment for age and baseline diameter. These findings highlight the need for larger, prospective studies to clarify the prognostic value of aortic deformation indices and their potential in risk stratification and clinical management of ATAA.

## Conflict of Interest Statement

The authors reported no conflicts of interest.

The *Journal* policy requires editors and reviewers to disclose conflicts of interest and to decline handling or reviewing manuscripts for which they may have a conflict of interest. The editors and reviewers of this article have no conflicts of interest.
